# The Position of the School Doctor

**Published:** 1913-08-16

**Authors:** 


### THE POSITION OF THE SCHOOL DOCTOR.

### The Board of Education Supports "The Hospital."

In a special article published in The Hospital of January 18, dealing with the advisability of establishing school clinics in London, it was urged that: "Medical inspection in the county is now second to none in the kingdom, but medical treatment still leaves much to be desired. Is it too much to hope that the Council will utilise the splendid material it has gathered together in its whole-time staff, and the admittedly excellent Public Health Department which it possesses, in dealing with this question of treatment? We think it will be agreed that a school doctor who confines his attention to inspection must deteriorate, however much he may keep up his knowledge by reading, unless he is given periodical opportunities to perfect himself in treatment. It is in the nature of his duties to report on treatment and its results; he is held to be a competent judge of the adequacy or otherwise of whatever treatment is given. Yet under present arrangements he is not given a chance of that practice which alone can make him perfect, and which will tend to broaden his activities and to enlarge his experience. In our view this is a mistake, and we feel that the Council will realise that it is not in the best interests of either the Department or the schools wholly to divorce inspection from treatment."

The Board's Letter.

This criticism has now been fully supported by the Board of Education in a letter on the subject which they have addressed to the London County Council. The letter states that: "It appears to the Board desirable that the Council should consider the possibility of extending the functions of the assistant medical officers. In the opinion of the Board there is a serious danger that unless more variety is introduced into the work of these officers, the restriction of their duties, in practice largely to routine medical inspection, re-examinations, and special examinations, will have an injurious influence upon the work generally. At the present time the quality of the Council's staff of whole-time assistant medical officers appears to be high. It seems probable, however, that if the existing limitations were to be maintained, either the enthusiasm of the medical officers for their work would diminish, with a resultant loss of efficiency, or there would be a tendency for the medical officers to seek employment in areas where a wider scope for their activities presented itself. With a view to securing greater variety, I am to suggest that in addition to carrying out the work of routine medical inspection in his district, together with re-examinations and special examinations, each assistant medical officer should be entrusted with other kinds of work, as, for example, the further examination of visual defects, Public Health work, including investigations of infectious diseases in schools, the medical inspection of children in secondary schools, scholarship candidates, etc., work in connection with the special schools, research and laboratory work, and some share in the working of the administrative arrangements."

The Education Committee of the Council are now considering whether it is possible to extend the functions of the assistant medical officers on the lines suggested.

